# Force‐Vector Pilates Exercises on Functional Performance and Braking Reaction Time in Older Professional Drivers: An Exploratory Feasibility Study

**DOI:** 10.1002/pri.70284

**Published:** 2026-08-01

**Authors:** Caio Cezar de Lima Maciel, Alexandre Leopold Busse, Ana Carolina Sauma Maluly, Angelica Castilho Alonso, Fernanda Botta Tarallo Rogatto, Matheus Henrique dos Santos Lino, Wilson Jacob Filho, Julia Maria D'Andréa Greve

**Affiliations:** ^1^ Graduate Program in Experimental Physiopathology, Faculdade de Medicina Universidade de Sao Paulo (FMUSP) Sao Paulo Brazil; ^2^ Laboratory of Medical Investigation in Aging (LIM‐66) Division of Geriatrics Hospital das Clínicas, Faculdade de Medicina, Universidade de Sao Paulo (FMUSP) Sao Paulo Brazil; ^3^ Laboratory of Movement Study Instituto de Ortopedia e Traumatologia do Hospital das Clínicas (IOT‐HC), Faculdade de Medicina FMUSP, Universidade de Sao Paulo Sao Paulo Brazil

**Keywords:** aging, braking reaction time, functional performance, physical therapy modalities, pilates‐based exercise

## Abstract

**Background and Purpose:**

Evidence regarding driving‐related outcomes in older professional drivers remains limited. This exploratory feasibility study aimed to evaluate the feasibility of implementing a force‐vector–oriented Mat Pilates program in older professional drivers and to explore potential changes in functional performance and braking reaction time.

**Methods:**

A single‐group prospective longitudinal study was conducted among older professional drivers (*n* = 49 eligible; *n* = 47 initiated intervention). Participants attended one supervised 50‐min Mat Pilates session per week for 30 sessions. Assessments were performed at baseline (M1), after 15 sessions (M2), and after 30 sessions (M3). Outcomes included TUG, handgrip strength, cervical rotation, anterior reach, SPPB, five‐repetition calf‐raise time (s) endurance, and braking reaction time in a driving simulator. Repeated measure comparisons across M1, M2, and M3 were performed using the Friedman test, with Kendall's W reported as effect size. Analyses were conducted per protocol.

**Results:**

Sixteen of 47 participants who initiated the intervention completed it (34.0%), with attrition primarily related to financial and logistical constraints. Statistically significant differences across time points were observed in mobility (TUG), lower‐limb performance (SPPB) 4‐m walking speed (m/s) and five‐repetition calf‐raise time (s), with lower times indicating better performance), cervical rotation (left), and anterior reach (*p* < 0.05). Handgrip strength showed no significant change. Braking reaction time showed a non‐significant numerical reduction from 0.90 ± 0.11s at M1 to 0.83 ± 0.15s at M3 (*p* = 0.098). Exploratory findings were derived from a small per‐protocol sample (*n* = 16) and should not be interpreted as evidence of intervention benefit.

**Conclusions:**

The force‐vector‐oriented Mat Pilates program was feasible to implement within this occupational context. Exploratory analyses identified changes in selected functional performance measures, including mobility and lower‐limb performance; however, these findings should not be interpreted as evidence of effectiveness due to the single‐group design, small per‐protocol sample, and absence of a control group. No statistically significant change was observed in the braking reaction time.

## 1 Introduction

The global aging process has led to an increase in the number of older drivers worldwide, reinforcing the importance of strategies aimed at maintaining functional independence and safe driving capacity among this population (Anstey et al. 2005; World Health Organization 2023). Age‐related declines in muscle strength, balance, flexibility, and reaction time are common and may compromise the ability of older adults to respond efficiently to complex motor demands, including those required during driving tasks (Marmeleira et al. 2009; Li et al. 2016). Physical exercise is associated with improvements in physical function and mobility in older adults, supporting its role in preserving neuromuscular performance and autonomy (Garber et al. 2011; Niyazi et al. 2024).

Among the available interventions, the Pilates Method, particularly its mat‐based form (Mat Pilates), has been widely applied in exercise and rehabilitation programs due to its emphasis on integrated muscle activation, posture control, and breathing coordination (Wells et al. 2012; Latey 2001; Kloubec 2010). Evidence from Pilates‐based exercise studies indicates improvements in neuromuscular coordination and functional performance, which may relate to the multidirectional motor demands encountered in daily activities (Carpes et al. 2008).

However, evidence remains limited regarding whether Pilates interventions, particularly when structured with a biomechanical emphasis on directional force vectors, are associated with driving‐related outcomes such as braking reaction time, a performance measure relevant to safe driving in older adults (Fujita et al. 2021; L. C. Pereira et al. 2022). In the present study, directional force vectors were used as a conceptual framework for exercise execution and progression rather than as directly measured biomechanical variables. The framework emphasizes how movement is performed through the intentional directional force strategies designed to promote postural organization, alignment, movement control, and body awareness during exercise execution. Accordingly, the proposed approach should be interpreted as an analytical and instructional model rather than a distinct Pilates method. Although theoretical relationships between movement control, neuromuscular coordination, and driving‐related performance have been proposed, direct evidence linking force‐vector–oriented Pilates interventions to braking reaction time remains limited. Therefore, the relationship investigated in the present study should be considered exploratory and hypothesis‐generating. Accordingly, the primary aim of this exploratory feasibility study was to evaluate recruitment, retention, adherence, safety, and intervention fidelity in older professional drivers. A secondary exploratory objective was to explore preliminary changes in functional performance and braking reaction time.

## 2 Material and Methods

### 2.1 Study Design and Participants

This study was designed as a single‐group, prospective, longitudinal exploratory feasibility study with repeated measures, conducted in insufficiently active older professional drivers. Physical inactivity was defined based on self‐report as engaging in less than 150 min per week of moderate‐intensity physical activity, in accordance with international physical activity guidelines. The primary objective was to assess feasibility metrics (recruitment, retention, adherence, safety, and intervention fidelity). A secondary objective was to explore potential changes in functional performance among participants who completed the intervention. This study was not designed to determine intervention effects or establish causal relationships. Consequently, any changes observed across assessment time points should be interpreted as exploratory and hypothesis‐generating rather than attributable to the intervention itself.

**FIGURE 1:**
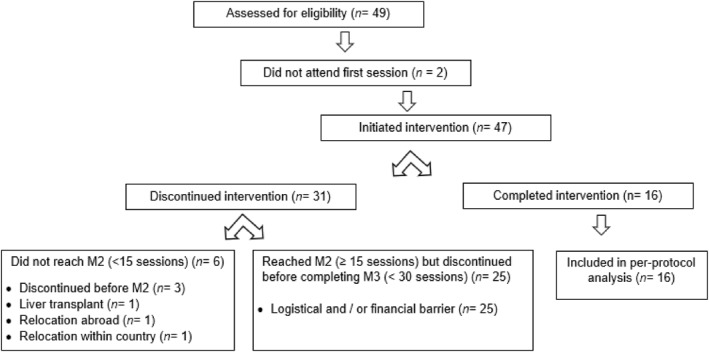
Flow of participants through the study.

A total of 49 eligible participants were assessed at baseline. Of these, 47 initiated the intervention, and 16 completed the full 30‐session protocol and were included in the final per‐protocol statistical analyses. The participant flow and attrition details are presented in Figure 1.

Participants were recruited through open invitations at urban taxi stands, dissemination via local driver associations, social media announcements, and snowball sampling (participant referral). Enrollment was voluntary, without financial incentives or mandatory participation. Eligibility was determined through structured interview and functional screening at baseline.

The occupational group was predominantly male. Although female drivers were present in the broader workforce, those meeting the age inclusion criterion (≥ 60 years) were exclusively male. Therefore, the final analytical sample consisted exclusively of men.

Inclusion criteria were: age ≥ 60 years, no history of fractures within the previous 3 months; ability to climb and descend one flight of stairs without assistance, and engaging in less than 150 min per week of moderate‐intensity physical activity at the time of enrollment. Exclusion criteria included any self‐reported condition that would prevent safe participation in physical testing or Pilates exercises as well as participation in any regular structured exercise program at the time of enrollment.

As this was an exploratory feasibility study, no formal a priori sample size calculation based on intervention effectiveness was performed. The target sample was defined pragmatically according to recruitment feasibility, availability of eligible older professional drivers, and the operational capacity to deliver a supervised 30‐session intervention. This approach is consistent with the purpose of pilot and feasibility studies, in which the sample is primarily intended to estimate recruitment, retention, adherence, safety, and procedural feasibility rather than to test intervention efficacy. The final analytical sample was determined by recruitment feasibility and participant retention throughout the intervention period.

All participants provided written informed consent. The study was approved by the Research Ethics Committee of the Hospital das Clinicas, Faculdade de Medicina, Universidade de Sao Paulo (HC‐FMUSP) (ethics approval n° 4.010.941) and registered in the Brazilian national ethics database, Plataforma Brasil. Following ethics approval in May 2020, recruitment was postponed because of the COVID‐19 pandemic and the temporary suspension of in‐person research activities at the study institution. Recruitment commenced in January 2022 after institutional authorization to safely resume face‐to‐face research. The first study intervention session was delivered on February 16, 2022. Recruitment remained open until January 2024, when enrollment was formally closed to allow all enrolled participants sufficient time to complete the planned 30‐session intervention within the study timeline. The intervention period concluded on November 27, 2024, when the final study intervention session was completed.

### 2.2 Assessments and Reassessments

All assessments were conducted by the same trained physiotherapist, blinded to the intervention phase, to ensure consistency and reduce measurement bias. Evaluations were performed at baseline (M1), 48 h after completion of the 15th session (M2), and 48 h after completion of the 30th session (M3). All assessments were conducted under standardized conditions, at the same time of day for each participant, and in a fixed testing order. Participants completed a brief familiarization trial for all performance‐based tests prior to formal data collection to minimize potential learning effects. Each assessment session lasted approximately 90 min.

Timed Up and Go (TUG) was performed twice, and the best time (seconds) was recorded. Handgrip strength was measured using a Jamar hydraulic dynamometer; two trials were performed for each hand (dominant and non‐dominant), and the highest value (kgf) was retained. Active cervical rotation (right and left) was measured using a goniometer with two trials per side and the highest value (degrees) recorded. The Functional Reach Test was performed twice, and the best distance (cm) was considered for analysis. The Short Physical Performance Battery (SPPB) was administered according to the original standardized protocol (0–12 scoring system), including balance testing, 4‐m walking speed (m/s), and the five‐times sit‐to‐stand test. For the five‐repetition calf‐raise time (s) endurance test, participants performed five bilateral heel raises as quickly as possible, and total execution time (seconds) was recorded.

Braking reaction time was assessed using a three‐dimensional driving simulator (Car Simulator Trainer F12PT, Foerst GmbH). After a familiarization trial in a traffic‐free virtual scenario, nine randomly presented visual “stop” stimuli were administered during the driving task. Participants were instructed to press the brake pedal with the right foot as quickly as possible. Braking reaction time (milliseconds) was defined as the interval between stimulus presentation and brake activation, and the mean of the final five trials was used for analysis. The system has been previously used in studies investigating braking reaction time and driving performance in older adults (Alonso et al. 2016; Canonica et al. 2023), supporting its applicability in this population.

### 2.3 Intervention Protocol

The intervention consisted of 30 classical Mat Pilates sessions, each lasting approximately 50 min, delivered once weekly over approximately 30 weeks (approximately 7–8 months).

A once‐weekly intervention frequency was intentionally selected to provide a pragmatic and feasible exercise schedule for older professional drivers who remained actively employed during the post‐pandemic period. Because most participants continued working as their primary source of household income, increasing the weekly training frequency would likely have reduced participation and long‐term adherence. Therefore, a supervised once‐weekly program was considered the most feasible schedule within the participants' real‐world occupational context. Consistent with the exploratory feasibility design of this study, the objective was to evaluate the feasibility of implementing this pragmatic intervention schedule rather than to determine the optimal training frequency for maximizing intervention efficacy.

The sessions were conducted in a group‐based format with up to 10 participants per class and were supervised by the same certified physiotherapist trained in the classical Pilates method to ensure procedural consistency. No assistant instructors were involved.

Each session followed a standardized structure: approximately 5 min of preparation and warm‐up, 40 min of exercise sequence, and 5 min of cool‐down and return to baseline. Transitions between exercises were brief (≤ 3 s), maintaining a continuous movement flow unless modification or rest was required to ensure safety or technique quality. Each exercise was performed as a single set of eight controlled dynamic repetitions, emphasizing precision, postural alignment, and breathing coordination. Progression was based on movement quality and absence of compensatory patterns rather than increases in exercise volume.

Exercises were performed in supine, prone, side‐lying, seated, kneeling, and standing positions. A total of 21 classical Mat Pilates exercises were implemented throughout the program (Table S1). Detailed descriptions of force‐vector organization, progression, regression strategies, and safety considerations are provided in Tables [Link], [Link].

The program was structured into three progressive blocks: Block 1 (Sessions 1–10), focused on familiarization and foundational neuromotor control; Block 2 (Sessions 11–20), characterized by increased movement complexity and multi‐planar integration; and Block 3 (Sessions 21–30), emphasizing refinement of motor control, postural integration, and execution precision. Progression occurred when participants were able to perform exercises with correct technique and without adverse symptoms. When necessary, modifications included reduced lever arms, decreased range of motion, simplified coordination demands, or additional external support.

Progression across blocks involved gradual increases in movement amplitude, lever arm length, multiplanar integration, and reduction of external support, while maintaining the same repetition structure.

Intensity was maintained at a moderate perceived effort level and regulated through qualitative movement criteria, including preservation of postural alignment, absence of compensatory patterns, controlled breathing, and the ability to complete all repetitions without symptom exacerbation. The protocol emphasized technique‐driven progression rather than fatigue‐based overload.

Participants were instructed to respect individual limits and immediately report pain, dizziness, or discomfort. Verbal cueing was continuously provided to distinguish expected muscular effort from potentially adverse symptoms. Safety monitoring occurred throughout each session, and exercises were modified or discontinued when necessary.

Attendance was recorded at every session to monitor adherence. Participants progressed to the second reassessment (M2) only after completing 15 sessions and to the third reassessment (M3) after completing all 30 sessions, as described in Section 2.2.

This study was reported in accordance with the CONSORT extension for pilot and feasibility trials. Given the single‐group repeated‐measures design, elements of the TREND statement were also considered to enhance reporting transparency.

### 2.4 Statistical Analysis

Data normality was assessed using the Shapiro–Wilk test. Descriptive statistics are presented as mean ± standard deviation (SD) for descriptive purposes and as median (interquartile range [IQR]) for inferential analyses.

Given the small sample size and the non‐normal distribution observed for most variables, nonparametric tests were applied for repeated‐measures comparisons. Differences across the three assessment time points (M1, M2, and M3) were analyzed using the Friedman test. When a significant global effect was identified, post hoc pairwise comparisons were conducted using the Wilcoxon signed‐rank test.

Effect size for the Friedman test was estimated using Kendall's coefficient of concordance (Kendall's *W*), interpreted as weak (< 0.3), moderate (0.3–0.5), or strong (> 0.5).

Ninety‐five percent confidence intervals (95% CI) for paired differences were calculated to estimate the magnitude and precision of changes across time points.

Detailed descriptive statistics, pairwise comparisons, and corresponding 95% confidence intervals are presented in Table S4.

The overall significance level for global (Friedman) tests was set at *p* < 0.05. To control for multiple pairwise comparisons within each outcome, Bonferroni correction was applied, and the adjusted significance level was set at *α* = 0.017 (0.05/3).

Given the exploratory and feasibility design and the number of outcomes analyzed, no formal multiplicity correction was applied across different outcomes. Therefore, *p*‐values should be interpreted cautiously.

## 3 Results

### 3.1 Feasibility Outcomes

The primary outcomes were feasibility metrics, including recruitment rate, retention, adherence, safety, and intervention fidelity. Secondary exploratory outcomes included functional performance measures and braking reaction time.

Of the 49 participants assessed for eligibility, 47 initiated the intervention (recruitment rate: 95.9%). Sixteen participants completed all 30 sessions and were included in the final per‐protocol analysis, resulting in a retention rate of 34.0% among initiators. Participant flow is presented in Figure 1. Although recruitment was successful, the retention rate was relatively low, indicating potential challenges for long‐term participation in this occupational population.

Attrition occurred primarily during the first half of the intervention and was mainly related to logistical and occupational constraints, including travel time to the intervention site and incompatibility with work schedules. No withdrawals were attributed to adverse events, and no serious adverse events were reported during the study.

Attendance was recorded at every session to monitor adherence. Among participants who completed the intervention, attendance was 100%, as completion of all 30 sessions was required for inclusion in the final per‐protocol analysis. However, overall study retention was 34.0%, indicating that adherence among completers should not be interpreted as reflecting participation across the entire recruited sample.

The intervention was delivered by the same certified physiotherapist following a predefined standardized protocol to promote procedural fidelity.

No participants discontinued the intervention due to intolerance to the exercises. Reported withdrawals were related to logistical barriers rather than dissatisfaction with the intervention. Based on participant self‐reports, no engagement in structured exercise outside the intervention, occupational workload changes, or major health events were identified during the study period.

### 3.2 Participant Characteristics

Baseline characteristics are presented for participants included in the per‐protocol analysis (*n* = 16). A total of 16 older taxi drivers (mean ± SD: 64.8 ± 3.3 years; BMI: 27.2 ± 2.1 kg/m^2^) completed the 30‐sessions Pilates intervention. Most participants self‐identified as White (62.5%), were married (81.3%), and had completed secondary education (43.8%). Half of the participants reported working 10–12 h per day, and 43.8% worked five days per week. Overall, 87.5% reported some degree of pain or physical limitation at baseline. Detailed baseline characteristics are presented in Table 1.

**TABLE 1:** Characteristics of the study participants (*n* = 16).

Characteristic	*n* (%)/Mean ± SD
Age (years)	64.8 ± 3.3
BMI (kg/m^2^)	27.2 ± 2.1
Skin color (white)	10 (62.5%)
Marital status (married)	13 (81.3%)
Education (secondary school completed)	7 (43.8%)
Working hours/day (10–12 h)	8 (50.0%)
Working days/week (5 days)	7 (43.8%)
Pain or physical limitation	14 (87.5%)
Hypertension	4 (25%)
Diabetes mellitus	4 (25%)
Epilepsy	0 (0%)

*Note:* Descriptive data of older taxi drivers included in the study.

### 3.3 Functional Outcomes

Global comparisons across the three assessment time points (M1, M2, and M3) using the Friedman test revealed statistically significant differences for TUG (*p* = 0.002; Kendall's *W* = 0.396), SPPB 4‐m walking speed (m/s) (*p* = 0.002; Kendall's *W* = 0.396), SPPB five‐times sit‐to‐stand (*p* = 0.009; Kendall's *W* = 0.297), cervical rotation (left) (*p* = 0.005; Kendall's *W* = 0.333), anterior reach (*p* = 0.025; Kendall's *W* = 0.231), and five‐repetition calf‐raise time (s) performance (*p* < 0.001; Kendall's *W* = 0.834). For time‐based outcomes, lower values represent better performance.

No significant global effects were observed for SPPB total score (*p* = 0.078), cervical rotation (right) (*p* = 0.185), handgrip strength (right: *p* = 0.150; left: *p* = 0.709), or braking reaction time (*p* = 0.269).

Post hoc pairwise comparisons using the Wilcoxon signed‐rank test with Bonferroni‐adjusted *α* (0.017) indicated differences primarily between M1 and M3 for TUG, SPPB 4‐m walking speed (m/s), SPPB five‐times sit‐to‐stand, cervical rotation (left), and five‐repetition calf‐raise time (s) performance. For anterior reach, a significant pairwise difference was observed between M2 and M3. Not all pairwise comparisons remained significant after adjustment.

Braking reaction time showed numerical variation across assessment moments; however, no statistically significant differences were identified in global or adjusted pairwise analyses. The 95% confidence intervals for paired differences are presented in Table 2. All analyses were conducted using complete‐case data from participants who completed the intervention (per‐protocol approach).

**TABLE 2:** Main functional outcomes across the three assessment moments (*n* = 16).

Variable	Baseline	15 sessions	30 sessions		
	(M1)	(M2)	(M3)	(*p*‐value Friedman's test)	Kendall's W
TUG (s)					
Median (IQR)	6.25 (4.98–8.34)	5.39 (5.04–7.11)	5.19 (4.84–5.61)	0.002	0.396
SPPB 4‐m walking speed (m/s)					
Median (IQR)	0.59 (0.51–0.84)	0.56 (0.46–0.64)	0.52 (0.47–0.55)	0.002	0.396
SPPB sit to stand 5x (s)					
Median (IQR)	11.84 (9.82–13.51)	9.84 (8.02–11.19)	8.99 (8.30–9.05)	0.009	0.297
SPPB (total score)					
Median (IQR)	11.50 (10–12)	12 (11.80–12)	12 (11.83–12)	0.078	0.160
Cervical rotation ‐right (°)					
Median (IQR)	56.40 (50–62.50)	60 (54.80–63.50)	60.2 (52.50–65.42)	0.185	0.105
Cervical rotation—left (°)					
Median (IQR)	60 (51–60)	65 (60–70)	65.42 (60–70)	0.005	0.333
Anterior reach (cm)					
Median (IQR)	33.84 (28.50–39.50)	31.10 (28–34.50)	37.25 (34.50–38.50)	0.025	0.231
Five‐repetition calf‐raise time (s)					
Median (IQR)	5.45 (4.79–7.48)	3.92 (2.83–4.58)	3.39 (2.59–3.78)	< 0.001	0.834
Handgrip‐right hand (kgf)					
Median (IQR)	41.91 (36.50–47)	41.27 (38–44.50)	44.29 (41.75–49)	0.150	0.119
Handgrip‐left hand (kgf)					
Median (IQR)	41.48 (34–48)	41.78 (36.95–48)	43.25 (41.50–47.50)	0.709	0.022
Braking time (s)					
Median (IQR)	0.90 (0.83–0.96)	0.84 (0.76–0.93)	0.82 (0.76–0.86)	0.269	0.082

*Note:* Values are presented as median (IQR). Global comparisons across the three assessment moments were performed using the Friedman test, with effect size expressed as Kendall's W. Pairwise comparisons were conducted using Wilcoxon signed‐rank tests with Bonferroni‐adjusted alpha (*α* = 0.017). *a* = M1 versus M2; *b* = M2 versus M3; *c* = M1 versus M3. SPPB = Short Physical Performance Battery. Lower values indicate better performance for TUG, SPPB 4‐m walking speed (m/s), SPPB sit‐to‐stand 5x, five‐repetition calf‐raise time (s), and braking time outcomes.

## 4 Discussion

This exploratory feasibility study demonstrated that implementation of a force‐vector‐based Mat Pilates protocol in older professional drivers was achievable, with favorable recruitment and safety indicators, although retention was limited. In exploratory secondary analyses, statistically significant differences across time points were observed in mobility (TUG), SPPB 4‐m walking speed (m/s), SPPB sit‐to‐stand performance, cervical rotation (left), and five‐repetition calf‐raise time (s) performance, whereas braking reaction time did not show statistically significant change. These findings indicate variation in selected functional performance measures but should not be interpreted as evidence of enhanced driving safety.

Retention represents an important feasibility consideration in the present study. Although recruitment and safety indicators were favorable, only 34% of participants who initiated the intervention completed the full protocol. This level of retention suggests that logistical and occupational barriers may substantially affect long‐term participation among older professional drivers. Future studies should consider alternative implementation strategies, including workplace‐based delivery models, flexible scheduling, or hybrid formats, to improve participant retention.

The use of a per‐protocol analytical approach introduces the possibility of attrition bias. Participants who completed the intervention may differ systematically from those who withdrew, potentially resulting in overestimation of intervention‐related effects. Therefore, the observed findings should be interpreted cautiously and viewed as exploratory.

Given the per‐protocol design and absence of a control group, causal inference cannot be established; therefore, the findings should be interpreted as exploratory and hypothesis‐generating.

The observed changes in TUG and SPPB components are consistent with randomized trials demonstrating the benefits of Pilates on balance and functional performance in older adults (Calvo‐Lobo et al. 2017). Enhanced cervical rotation and five‐repetition calf‐raise time (s) performance may be associated with changes in segmental mobility and lower‐limb muscular endurance, both relevant to postural control and general mobility.

Neurophysiological evidence indicates that coordinative training can induce spinal and supraspinal adaptations, improving sensorimotor integration and movement efficiency (Taube et al.). In the present study, the force‐vector framework was used as an instructional strategy to guide exercise execution and progression. The framework was not directly operationalized through biomechanical measurements and therefore cannot be interpreted as evidence of specific vector‐related mechanisms. Rather, it provided a structured approach for emphasizing directional force application during movement performance. In older adults, better physical performance has been associated with functional independence and mobility outcomes (Sakurai et al. 2021). Although braking reaction time showed numerical variation across assessments, it did not reach statistical significance (World Health Organization).

Alternative explanations should be considered equally plausible causes of the observed changes. Given the absence of a control group, the relative contribution of these alternative explanations cannot be distinguished from potential intervention‐related effects. Repeated exposure to simulator testing may have introduced a learning effect. Regression to the mean cannot be excluded given the small sample size. Seasonal or occupational workload variations may have influenced physical performance across time points. Additionally, participant awareness of being observed may have contributed to behavioral changes (Hawthorne effect).

The observed changes within a once‐weekly protocol demonstrate that outcome assessment was feasible and that measurable variation across time points could be detected. However, given the exploratory single‐group design, these findings should be considered hypothesis‐generating rather than evidence of intervention effects. Previous studies have suggested that physical performance measures may be responsive to exercise interventions in older adults and that Pilates‐based exercise may be associated with functional benefits in aging populations; however, direct comparisons should be interpreted cautiously given differences in study design, intervention dose, and participant characteristics (Sakurai et al. 2021; M. J. Pereira et al. 2022). In occupational contexts, such frequency may represent a feasible implementation strategy; nevertheless, adequately powered randomized controlled trials are required to evaluate effectiveness.

The operationalization of directional force vectors within Mat Pilates represents a conceptual biomechanical framework that may inform future research on exercise prescription, movement control, and functional performance in aging populations. Future investigations incorporating direct biomechanical assessments may help clarify whether specific directional force strategies are associated with distinct functional adaptations.

As the sample consisted exclusively of older male taxi drivers, generalization to other populations should be made cautiously (Sánchez et al. 2019).

## 5 Study Limitations

Baseline characteristics presented refer only to participants included in the per‐protocol analysis. Baseline data from participants who discontinued the intervention were incomplete and therefore could not be compared with those of the completers. Consequently, it was not possible to determine whether systematic differences existed between completers and non‐completers, increasing the potential risk of attrition bias.

This study has limitations that should be considered when interpreting the findings. The absence of a control group and randomization substantially restricts causal inference and prevents determination of whether observed changes resulted from the intervention, natural variation, learning effects, regression to the mean, or other external influences. The relatively small, male‐only sample may have reduced statistical power and limits generalizability to female or non‐occupational older adult populations.

Recruitment relied on voluntary participation through open invitations and snowball sampling, which may have increased the likelihood of volunteer bias and limited representativeness of the broader population of older professional drivers. Additionally, physical inactivity was classified based on self‐report rather than objective measurement, which may have introduced misclassification bias.

Although the sample composition reflects the demographic characteristics of older male taxi drivers—the target population of the intervention—it also constrains broader external validity. Variability in occupational workload across the study period may have influenced performance across assessment points.

Because multiple outcomes were analyzed, the possibility of Type I error due to multiple comparisons cannot be entirely excluded despite statistical adjustment. Furthermore, the relatively small sample size and exploratory design increase the possibility of a Type II error, particularly regarding braking reaction time. Although exposure to the simulator was brief and assessment intervals were extended, potential familiarization effects associated with repeated testing cannot be completely ruled out.

Accordingly, findings should be interpreted within the context of an exploratory feasibility design and require confirmation through adequately powered randomized controlled trials.

The relatively low retention rate and reliance on per‐protocol analyses further limit interpretation of the findings, as completers may not be representative of the originally recruited sample.

## 6 Conclusion

This exploratory feasibility study demonstrated that a force‐vector–oriented Mat Pilates protocol was feasible to implement in older professional drivers with favorable recruitment and safety indicators, although retention was limited.

Exploratory analyses identified changes in selected functional performance measures; however, these findings should be interpreted as preliminary and hypothesis‐generating rather than as evidence of intervention effects. Findings should be interpreted within the context of a single‐group exploratory design without a control group and with limited statistical power. Interpretation should also consider the relatively low retention rate and the potential for attrition bias associated with the per‐protocol analytical approach.

The potential role of directional force‐vector application in driving‐related performance remains to be established. These findings support the rationale for future adequately powered randomized controlled trials designed to evaluate the clinical relevance and underlying mechanisms of force‐vector–oriented Pilates interventions in aging populations.

## 7 Implications for Future Research

This exploratory feasibility study primarily informs the design of future research rather than clinical practice. The findings support further investigation of force‐vector–oriented Pilates interventions in adequately powered randomized controlled trials designed to evaluate whether observed changes can be confirmed under controlled conditions, implementation feasibility, and potential mechanisms in aging populations. The force‐vector framework may provide a conceptual biomechanical model for future studies examining movement control and functional performance in older adults.

## Author Contributions

**Caio Cezar de Lima Maciel:** principal author, study conception, text elaboration, and intervention execution. **Alexandre Leopold Busse:** study supervision, study design, and manuscript revision. **Ana Carolina Sauma Maluly:** data collection, evaluation. **Angelica Castilho Alonso:** study design, evaluation, manuscript revision. **Fernanda Botta Tarallo Rogatto:** data collection, evaluation. **Matheus Henrique dos Santos Lino:** data collection, evaluation. **Wilson Jacob Filho:** study design, manuscript revision. **Julia Maria D'Andréa Greve:** study design, manuscript revision.

## Funding

The authors have nothing to report.

## Ethics Statement

The study was conducted after approval by the Research Ethics Committee of the Hospital das Clínicas, Faculdade de Medicina, Universidade de Sao Paulo (HC‐FMUSP) (protocol 4.010.941) and registration in Plataforma Brasil.

## Consent

All participants provided written informed consent prior to participation.

## Conflicts of Interest

The authors declare no conflicts of interest.

## Supporting information


**Table S1:** Classical Mat Pilates exercises included in the intervention.

**Table S2:** Structured Mat Pilates exercises with tridimensional force‐vector application.

**Table S3:** Progression structure and neuromotor and safety emphasis of the classical Mat Pilates exercises.

**Table S4:** Detailed pairwise comparisons and descriptive statistics for functional outcomes across the three assessment moments (*n*=16).

## Data Availability

The data that support the findings of this study are available on request from the corresponding author. The data are not publicly available due to privacy or ethical restrictions.
