# Supplementary material for: Force‐Vector Pilates Exercises on Functional Performance and Braking Reaction Time in Older Professional Drivers: An Exploratory Feasibility Study
Source: Physiother Res Int. 2026 Aug 1;31(4):e70284. doi: 10.1002/pri.70284 (PMC13428497; doi:10.1002/pri.70284)
Supplement: Supplementary file 1 — Table S1: Classical Mat Pilates exercises included in the intervention. [file PRI-31-e70284-s003.docx]

**Supplementary Table S1.** *Classical Mat Pilates exercises included in the intervention*

| **Exercise name** |
| --- |
| The hundred |
| The roll up |
| The one leg circle |
| Rolling back |
| The one leg stretch |
| The double leg stretch |
| The spine stretch |
| The Saw |
| The swan dive |
| The one leg kick |
| The double kick |
| The neck pull |
| The shoulder bridge |
| The spine twist |
| The side kick |
| Swimming |
| The leg pull – front |
| The leg pull – back |
| The side kick kneeling |
| The side bend |
| The push up |

**Note:** List of 21 classical Mat Pilates exercises performed during the intervention*.*
