# Supplementary material for: Force‐Vector Pilates Exercises on Functional Performance and Braking Reaction Time in Older Professional Drivers: An Exploratory Feasibility Study
Source: Physiother Res Int. 2026 Aug 1;31(4):e70284. doi: 10.1002/pri.70284 (PMC13428497; doi:10.1002/pri.70284)
Supplement: Supplementary file 2 — Table S2: Structured Mat Pilates exercises with tridimensional force‐vector application. [file PRI-31-e70284-s002.docx]

**Supplementary Table S2.** *Structured Mat Pilates exercises with tridimensional force-vector application*

| **Exercise** | **Starting position** | **Tridimensional force-vector direction** |  |
| --- | --- | --- | --- |
| The Hundred | Supine, legs tabletop, arms extended | Axial opposition (spine lengthens); heels press down while hands project forward/up. |  |
| The Roll Up | Supine, arms overhead | Axial vector with opposition between heels (forward) and hands (forward/up); pure segmental flexion. |  |
| One Leg Circle | Supine, one leg raised | Axial support; active leg opposes anchored heel; pelvic control in three planes. |  |
| Rolling Back | Seated, Knees flexed, hands on shins | Axial + anteroposterior opposition; segmental roll with centered control. |  |
| One Leg Stretch | Supine | Axial support; alternating anteroposterior opposition between legs; pelvis stable. |  |
| Double Leg Stretch | Supine | Cross-diagonal opposition of arms and legs integrated with axial alignment. |  |
| Spine Stretch Forward | Supine, arms/ legs extend | Axial elongation; anteroposterior opposition (fingertips forward / sit bones down). |  |
| The Saw | Seated, legs extended | Axial support with spiral rotation; hands in opposite direction (one forward, one back). |  |
| Swan Dive | Seated, legs apart | Axial + anteroposterior opposition (sternum forward/up; legs back) with thoracic expansion. | |
| One Leg Kick | Prone, forearms on mat | Axial support; opposition between active heel (up/back) and forearms (down). |  |
| Double Kick | Prone, hands clasped | Axial + anteroposterior opposition; controlled thoracic extension. |  |
| Neck Pull | Supine, hands behind head | Axial vector; opposition heels forward / crown forward; segmental flexion. |  |
| Shoulder Bridge | Supine, Knees flexed | Axial elongation; feet press down while pelvis lifts (anteroposterior opposition). |  |
| Spine Twist | Seated, legs extended | Axial support with controlled axial rotation (spiral) of trunk. |  |
| Side Kick | Side-Lying, lower arm extended | Axial support; bottom leg presses down while top leg reaches away (lateral opposition). |  |
| Swimming | Prone, alternating limbs | Cross-diagonal opposition of limbs with axial support; alternating coordination. |  |
| Leg Pull – Front | Plank | Axial + anteroposterior opposition (hands/feet counter-press) maintaining straight line. |  |
| Leg Pull – Back | Reverse Plank | Axial + anteroposterior opposition (hands down / heels away) with posterior expansion. |  |
| Side kick Kneeling | Kneeling on one Knee/ hand | Axial support; lateral/diagonal opposition between support and moving limb. |  |
| Side Bend | Side support | Axial + lateral opposition; controlled trunk lift forming a three-dimensional arc. |  |
| Push Up | Stand to plank | Axial + anteroposterior opposition; whole-body alignment during descent/ascent. |  |

**Note:** All exercises were performed once per week within 50-minute sessions across 30 sessions. Each exercise was executed for 8 repetitions in one set, with qualitative progression focused on control, coordination, and tridimensional stability.
