# Supplementary material for: Force‐Vector Pilates Exercises on Functional Performance and Braking Reaction Time in Older Professional Drivers: An Exploratory Feasibility Study
Source: Physiother Res Int. 2026 Aug 1;31(4):e70284. doi: 10.1002/pri.70284 (PMC13428497; doi:10.1002/pri.70284)
Supplement: Supplementary file 3 — Table S3: Progression structure and neuromotor and safety emphasis of the classical Mat Pilates exercises. [file PRI-31-e70284-s004.docx]

**Supplementary Table S3.** *Progression structure and neuromotor and safety emphasis of the classical Mat Pilates exercises*

| **Exercise** | **Qualitative progression**  **(levels 1–3)** | **Neuromotor and safety emphasis** | |
| --- | --- | --- | --- |
| The Hundred | Level-1 Feet on floor; Level-2 Tabletop;  Level-3 Legs extended | Maintain cervical alignment and breath-core coupling to prevent strain. | |
| The Roll Up | Level-1 Hands on thighs assist; Level-2 Arms extended; Level-3 Smooth segmental roll | Control segmental flexion to maintain cervical neutrality and trunk alignment. | |
| One Leg Circle | Level-1 Small circles; Level-2 Increased range; Level-3 Stable pelvis | Maintain pelvic stability and control hip circumduction. | |
| Rolling Back | Level-1 Short range; Level-2 Full roll;  Level-3 Smoother control | Coordinate breath timing to ensure cervical safety and smooth. | |
| One Leg Stretch | Level-1 Short reach; Level-2 Full reach;  Level-3 Tempo control | Maintain pelvic stability with core engagement. | |
| Double Leg Stretch | Level-1 Reduced range; Level-2 Full extension;  Level-3 Hold control | Control lumbar extension through synchronized breathing. | |
| Spine Stretch Forward | Level-1 Short flexion; Level-2 Increased reach;  Level-3 Segmental flexion | Maintain axial elongation and control flexion to improve segmental mobility. | |
| The Saw | Level-1 Small rotation; Level-2 Reach to foot; Level- 3 Longer, controlled spiral | Coordinate rotation and reach to enhance thoracic flexibility and trunk stability. | |
| Swan Dive | Level-1 Partial lift; Level-2 Thoracic extension; Level-3 Controlled rocking | Promote thoracic extension with posterior chain control and breath coordination. |  |
| One Leg Kick | Level-1 Small kicks; Level-2 Full kicks;  Level-3 Tempo control | Facilitate alternating leg action while maintaining pelvic alignment and lumbar stability. | |
| Double Kick | Level-1 Small kicks; Level-2 Thoracic lift; Level-3 Full pattern | Prevent hyperextension and promote smooth coordination between limbs and trunk. | |
| Neck Pull | Level-1 Hands assist; Level-2 Arms active; Level-3 Pure segmental flexion | Maintain cervical neutrality and segmental control during flexion. | |
| Shoulder Bridge | Level-1 Partial lift; Level-2 Full bridge;  Level-3 Added stability challenge | Maintain alignment to enhance thoraco-pelvic control. | |
| Spine Twist | Level-1 Small rotation; Level-2 Full rotation; Level-3 Tempo/breath control | Maintain pelvic stability and control rotation for axial alignment. | |
| Side Kick | Level-1 Small range; Level-2 Full range;  Level-3 Tempo control | Maintain lateral stability through contralateral opposition. | |
| Swimming | Level-1 Static hold; Level-2 Slow alternation; Level-3 Continuous alternation | Maintain trunk stability and coordinate limb alternation to prevent lumbar sway. | |
| Leg Pull – Front | Level-1 Static plank; Level-2 Small leg lift; Level-3 Controlled alternation | Maintain scapular support and trunk alignment during extension. | |
| Leg Pull – Back | Level-1 Static reverse plank; Level-2 Small heel reach; Level-3 Alternation | Maintain shoulder stability and neutral neck alignment. | |
| Side kick Kneeling | Level-1 Small range; Level-2 Full lateral reach; Level-3 Diagonal challenge | Maintain vertical trunk alignment and three-dimensional balance. | |
| Side Bend | Level-1 Partial lift; Level-2 Full arc;  Level-3 Time under tension | Promote shoulder stability and oblique activation with controlled breathing. | |
| Push Up | Level-1 Incline or knees; Level-2 Full plank; Level-3 Tempo control | Maintain scapular alignment and controlled descent to ensure cervical neutrality. | |

**Note**: All exercises were performed for eight repetitions in a single set. Progression followed three qualitative levels (Level 1: basic facilitation, Level 2: intermediate control, Level 3: near-final posture) without changes in training volume or external load. Each progression emphasized three-dimensional force-vector opposition to optimize postural control and neuromotor safety.
