# Supplementary material for: Force‐Vector Pilates Exercises on Functional Performance and Braking Reaction Time in Older Professional Drivers: An Exploratory Feasibility Study
Source: Physiother Res Int. 2026 Aug 1;31(4):e70284. doi: 10.1002/pri.70284 (PMC13428497; doi:10.1002/pri.70284)
Supplement: Supplementary file 4 — Table S4: Detailed pairwise comparisons and descriptive statistics for functional outcomes across the three assessment moments (n=16). [file PRI-31-e70284-s001.docx]

**Supplementary Table S4.** *Detailed pairwise comparisons and descriptive statistics for functional outcomes across the three assessment moments (n=16)*

| **Variable** | **Baseline** | | **15 sessions** | **30 sessions** |
| --- | --- | --- | --- | --- |
|  | **(M1)** | | **(M2)** | **(M3)** |
| TUG (s) |  | |  |  |
| Mean (SD) | 6.72 (1.98) | | 5.84 (1.22) | 5.19 (0.63) |
| p-value | 0.012^a*^ | | 0.010^b*^ | 0.020^c^ |
| 95%CI | 0.22 – 1.53 | | 0.20 – 1.11 | 0.61 – 2.45 |
| SPPB 4-m walking speed (m/s) |  | |  |  |
| Mean (SD) | 0.70 (0.27) | | 0.55 (0.11) | 0.52 (0.06) |
| p-value | 0.003^a*^ | | 0.155^b^ | 0.002^c*^ |
| 95%CI | 0.03 – 0.27 | | -0.01 – 0.09 | 0.05 – 0.31 |
| SPPB sit to stand 5x (s) |  | |  |  |
| Mean (SD) | 11.90 (2.99) | | 10.09 (3.27) | 9.05 (1.46) |
| p-value | 0.002^a*^ | | 0.148^b^ | 0.005^c*^ |
| 95%CI | 0.83 – 2.79 | | -0.44 – 2.51 | 1.24 – 4.45 |
| SPPB (total score) |  | |  |  |
| Mean (SD) | 11.00 (1.26) | | 11.60 (0.88) | 11.83 (0.33) |
| p-value | 0.016^a*^ | | 0.610^b^ | 0.033^c^ |
| 95%CI | -1.07 – -0.13 | | -0.72 – 0.26 | -1.54 – -0.13 |
| Cervical rotation -right (º) |  | |  |  |
| Mean (SD) | 57.80 (10.93) | | 59.60 (16.81) | 60.83 (10.14) |
| p-value | 0.569^a^ | | 0.624^b^ | 0.053^c^ |
| 95%CI | -7.49 – 3.89 | | -6.44 – 3.98 | -6.32 – 0.26 |
| Cervical rotation – left (º) |  | |  |  |
| Mean (SD) | 57.40 (7.68) | | 64.40 (6.72) | 65.42 (6.94) |
| p-value | 0.021^a^ | | 0.346^b^ | 0.002^c*^ |
| 95%CI | -12.35 – -1.65 | | -5.29 – 3.26 | -12.20 – -3.84 |
| Anterior Reach (cm) |  | |  |  |
| Mean (SD) | 33.67 (8.08) | | 31.20 (5.17) | 37.25 (5.38) |
| p-value | 0.196^a^ | | 0.005^b*^ | 0.066^c^ |
| 95%CI | -1.61 – 6.54 | | -9.86 – -2.24 | -7.33 – 0.17 |
| five-repetition calf-raise time (s) | |  |  |  |
| Mean (SD) | 5.89 (2.19) | | 4.34 (2.11) | 3.39 (1.02) |
| p-value | 0.001^a*^ | | 0.002^b*^ | 0.001^c*^ |
| 95%CI | 0.69 – 2.42 | | 0.29 – 1.61 | 1.56 – 3.44 |
| Handgrip-right hand (kgf) |  | |  |  |
| Mean (SD) | 41.81 (7.21) | | 40.73 (9.35) | 44.29 (6.60) |
| p-value | 0.513^a^ | | 0.116^b^ | 0.132^c^ |
| 95%CI | -2.38 – 4.54 | | -8.47 – 1.36 | -6.02 – 1.06 |
| Handgrip-left hand (kgf) |  | |  |  |
| Mean (SD) | 41.95 (9.06) | | 41.55 (9.08) | 43.25 (7.04) |
| p-value | 0.865^a^ | | 0.395^b^ | 0.437^c^ |
| 95%CI | -3.22 – 4.01 | | -6.00 – 2.61 | -3.83 – 1.22 |
| Braking time (s) |  | |  |  |
| Mean (SD) | 0.90 (0.11) | | 0.86 (0.14) | 0.83 (0.15) |
| p-value | 0.234^a^ | | 0.379^b^ | 0.098^c^ |
| 95%CI | -0.04 – 0.12 | | -0.04 – 0.09 | -0.03 – 0.16 |

**Note:** Values are presented as mean (SD). Pairwise comparisons were conducted using Wilcoxon signed-rank tests with Bonferroni-adjusted alpha (α = 0.017). a = M1 vs M2; b = M2 vs M3; c = M1 vs M3. SPPB = Short Physical Performance Battery. Lower values indicate better performance for TUG, SPPB 4-m walking speed (m/s), SPPB sit-to-stand 5x, five-repetition calf-raise time (s), and braking time outcomes.
